# Environmental distribution of Porcine Circovirus Type 2 (PCV2) in swine herds with natural infection

**DOI:** 10.1038/s41598-019-51473-6

**Published:** 2019-10-15

**Authors:** Gonzalo López-Lorenzo, José Manuel Díaz-Cao, Alberto Prieto, Cynthia López-Novo, Ceferino Manuel López, Pablo Díaz, Víctor Rodríguez-Vega, Pablo Díez-Baños, Gonzalo Fernández

**Affiliations:** 1 Department of Animal Pathology (INVESAGA Group), Faculty of Veterinary Sciences, Universidade de Santiago de Compostela, 27002 Lugo, Spain; 2 Boehringer Ingelheim Animal Health, Barcelona, Spain

**Keywords:** Environmental microbiology, Viral epidemiology

## Abstract

Porcine circovirus type 2 (PCV2) is the aetiological agent of PCV2-Systemic Disease (PCV2-SD) and PCV2-Subclinical Infection (PCV2-SI). PCV2 is highly resistant to environmental conditions, being able to remain in the farm environment and thus represent a risk for infection maintenance. The aim of this study was to identify, under field conditions, the possible critical points in the environment of non-vaccinated farrow-to-weaning swine farms where PCV2 could accumulate and persist. For that, environmental samples from five swine farms with PCV2-SD or PCV2-SI were taken and analysed by qPCR, including different farm areas, farm personnel and management implements. PCV2 DNA was detected in the environment of all farms (42.9% of positive samples). Overall, the PCV2-SD herd seemed to present more positive samples and higher viral loads than the PCV2-SI herds. At individual farm level, weaning areas appeared to be the most contaminated facilities. In addition, PCV2 was found at high levels in most samples from farm workers, especially work boots, suggesting that they may play a role in within-farm transmission. In addition, PCV2 was detected in areas without animals the like warehouses, offices and farm perimeter. Therefore, this study is helpful to improve measures to reduce within-farm PCV2 dissemination.

## Introduction

Porcine circovirus type 2 (PCV2), a non-enveloped DNA virus present in most porcine farms, is considered one of the most important pathogens for swine production worldwide^1^. The absence of an external envelope provides PCV2 a strong resistance to both chemicals and temperatures^2,3^. This fact suggests that PCV2 may be able to remain in the environment for extended periods, thus favouring the maintenance of the infection due to the contamination of different farm facilities.

This virus is linked to a variety of syndromes grouped together as porcine circovirus diseases (PCVDs)^4^. Among them, PCV2-systemic disease (PCVD-SD) represents the clinical presentation, with signs such as wasting, dyspnoea, enlarged lymph nodes, paleness of skin and diarrhoea^5^. However, the economic losses produced by PCV2-subclinical infection (PCV2-SI) at farm level are higher than the cost of the pigs affected by PCV2-SD, mainly due to the decrease in daily weight gain and vaccine effectiveness^6–8^.

PCV2 can infect pigs from one week of age to adult sows; however, in field conditions, the onset of the disease is usually detected in the weaning period^9,10^. Regarding the infection dynamics, differences between PCV2-SD and PCV2-SI have hardly been observed^11,12^, although viremia and PCV2 shedding are higher in PCV2-SD^13^. PCV2 is shed through secretions and excretions of infected pigs^14^, and direct contact between animals is considered the most efficient form of transmission^15^; however, indirect transmission is also thought to occur through contaminated vectors or fomites. Regarding this, studies carried out with other viral pathogens like Porcine Respiratory and Reproductive Syndrome Virus (PRRSV) or Influenza A virus have demonstrated the involvement of farm fomites and personnel in the within-farm transmission^16,17^. Nevertheless, no studies have focused on this hypothesis for PCV2, with the only exception of Dvorak *et al*.^18^ who found this virus in environmental samples from farrowing areas. Therefore, the possibility of PCV2 contamination has not been evaluated yet in other farm facilities, as well as in personnel and in the different elements involved in animal management. Considering the extreme resistance of PCV2 in the environment, the assessment of PCV2 contamination in the different farm areas, workers and other elements would be helpful in order to establish which of them pose a higher risk of acting as PCV2 reservoirs or vectors.

For the aforementioned reasons, the aim of this study was to evaluate, under field conditions, the environmental PCV2 contamination of different farm facilities, personnel and animal management implements in non-vaccinated swine herds with PCV2-SD and PCV2-SI, estimating the viral load by qPCR.

## Results

### Farms included in the study

Seven farrow-to-weaning swine farms, located in North-western Spain, met the criteria to be eligible for the study, and five of them agreed to participate. The characteristics of the studied farms are gathered in Table 1.

**Table 1:** Characteristics of the included farms.

	Farm A	Farm B	Farm C	Farm D	Farm E
PCV2 compatible clinical signs	No	No	No	No	Yes
Increased mortality rate at weaning age*	No	No	No	No	Yes
N° sows	175	500	200	500	125
N° buildings	1	5	4	4	1
N° farrowing rooms	3	8	3	8	2
N° weaning rooms	3	6	3	7	2
N° gestation rooms	2	1	2	1	1
Office	Yes	Yes	Yes	Yes	Yes
Warehouse area	Yes	No	Yes	Yes	Yes

*Mortality rate higher than 4% and also higher than mean of % historic mortality + 1.66 x standard deviation.

### PCV2 circulation confirmation and farm classification

Viral circulation was demonstrated in the five herds. Four of them (farms A-D) showed only one qPCR positive blood pool, with values ranging between 9.8 × 10^5^–1.7 × 10^6^ PCV2 copies/ml blood; in addition, none of these herds presented clinical signs compatible with PCV2 infection or an increase of post-weaning mortality, and for these reasons they were classified as PCV2-SI herds. In contrast, the remaining herd (Farm E) showed PCV2-compatible clinical signs and increased mortality at the post-weaning phase. In addition, all blood pools from it tested positive to qPCR (4.3 × 10^6^–9.3 × 10^8^ PCV2 copies/ml blood). Moreover, five dead pigs from this farm were necropsied on the sampling date. qPCR analysis of inguinal lymph nodes, spleen and lung demonstrated a high viral load for one animal (3.7 × 10^6^–3.5 × 10^7^ PCV2 copies/500 ng total DNA), and histopathological examination of this pig also showed PCV2-compatible lesions. Consequently, this farm was classified as PCV2-SD herd.

### PCV2 environmental distribution

A total of 66 out of 154 (42.9%) environmental samples were positive to PCV2 DNA. In PCV2-SI herds, the percentage of PCV2 positive environmental samples ranged from 15.6% in Farm D to 56.2% in Farm A. In the PCV2-SD herd (Farm E), this percentage reached 86.7%.

The results for each environmental sample type are summarized in Table 2. Overall, PCV2 contamination was detected in all types of environmental samples, except for the sow feeders and the feed silo rungs. PCV2 was frequently detected in samples from work boots, piglet hoppers, weaning pen walls and weaning corridors. Overall, the highest counts of PCV2 copies were detected in samples from the weaning areas; the highest value was detected in the pen floor from the PCV2-SD herd (Farm E), whereas in the PCV2-SI herds the highest amounts corresponded to pen railing in Farm A, air fan in Farm B, and pen wall in Farm C. Surprisingly, all samples from the weaning area in farm D were negative.

**Table 2:** Number of PCV2 copies/swab for each sample type in each herd.

	PCV2-SI herds				PCV2-SD herd
	Farm A	Farm B	Farm C	Farm D	Farm E
Farrowing area	Mean: 7.1 × 10^4^				Mean: 4.6 × 10^5^
Sow feeder	—	—	—	—	—
Sow crate	—	—	—	—	6.3 × 10^3^
Piglet resting area	—	—	—	—	7.6 × 10^2^
Farrowing corridor	7.1 × 10^4^	—	—	—	1.5 × 10^6^
Farrowing air fan	—	—	—	—	6.5 × 10^5^
Delivery management tool box	—	—	—	—	1.7 × 10^4^
**Weaning area**	**Mean: 2.7 × 10** ^**5**^				**Mean: 1.4 × 10** ^**7**^
Piglet hopper	5.9 × 10^4^	9.1 × 10^2^	6.0 × 10^5^	—	7.6 × 10^4^
Weaning pen wall	7.8 × 10^2^	7.8 × 10^2^	1.0 × 10^6^	—	1.4 × 10^6^
Weaning pen floor	—	—	1.1 × 10^5^	—	4.1 × 10^7^
Weaning corridor	4.9 × 10^5^	2.4 × 10^2^	1.4 × 10^5^	—	2.8 × 10^7^
Weaning pen railing	9.8 × 10^5^	—	6.9 × 10^4^	—	8.5 × 10^6^
Weaning air fan	—	3.5 × 10^5^	6.6 × 10^3^	—	3.3 × 10^6^
**Gestation area**	**Mean: -**				**Mean: 4.8 × 10** ^**5**^
Sow hopper	—	—	—	—	2.4 × 10^4^
Gestation pen floor	—	—	—	—	1.86 × 10^5^
Gestation corridor	—	—	—	—	1.2 × 10^6^
**Farm warehouse**	**Mean: 1.4 × 10** ^**5**^				**Mean: 1.8 × 10** ^**6**^
Warehouse floor	7.1 × 10^5^	Not sampled	—	1.0 × 10^4^	Not sampled
Working utensils	—	Not sampled	—	2.1 × 10^3^	1.5 × 10^6^
Feed wagons	2.0 × 10^3^	Not sampled	—	—	8.1 × 10^5^
Pressure washer	5.8 × 10^3^	Not sampled	—	—	1.3 × 10^6^
Sorting panel	9.3 × 10^4^	—	—	1.4 × 10^5^	3.6 × 10^6^
**Farm personnel**	**Mean: 2.0 × 10** ^**5**^				**Mean: 5.9 × 10** ^**5**^
Farmer hands	—	—	4.6 × 10^3^	—	1.1 × 10^5^
Nostrils	4.1 × 10^3^	—	—	8.5 × 10^3^	9.5 × 10^3^
Hair/Hat	2.2 × 10^3^	6.2 × 10^3^	—	—	4.8 × 10^4^
Workwear	2.3 × 10^5^	4.1 × 10^3^	—	—	4.9 × 10^5^
Work boots	2.9 × 10^5^	1.5 × 10^3^	1.4 × 10^6^	—	2.3 × 10^6^
**Office**	**Mean: 5.8 × 10** ^**3**^				**Mean: 5.2 × 10** ^**5**^
Office floor	1.7 × 10^4^	—	—	—	Not sampled
Door handles	3.1 × 10^3^	—	—	—	3.1 × 10^5^
Pen/Computer keyboard	1.7 × 10^3^	—	—	—	—
Tables/Chairs	5.0 × 10^3^	—	1.9 × 10^3^	—	7.2 × 10^5^
**Perimeter**	**Mean: 1.6 × 10** ^**3**^				**Mean: 3.8 × 10** ^**5**^
Parking area	4.2 × 10^3^	—	—	2.8 × 10^2^	-
Farm main entrance	—	3.4 × 10^2^	—	—	3.8 × 10^5^
Feed silo rung	—	—	—	—	—

“—” indicates a result below the limit of detection after PCV2 qPCR analysis using both DNA extraction methods.

Regarding the PCV2-SD herd, 100% of the samples from the weaning area, gestation area, farm warehouse and farm personnel were positive to PCV2 DNA. In the rest of the areas, the percentage of positive samples was: 83.3% in the farrowing area, 66.7% in the office and 33.3% in the farm perimeter. The mean of PCV2 copies/swab in the mentioned categories ranged from 1.4 × 10^7^ in the weaning area to 3.8 × 10^5^ in the perimeter.

**Figure 1:**
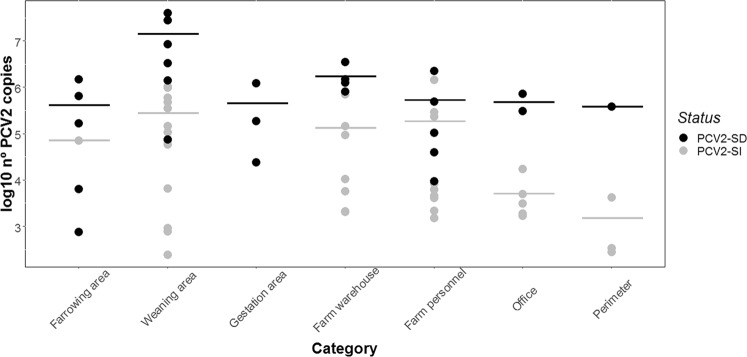
Graphical representation of log10 PCV2 copies per swab of positive samples from each environmental category. Black dots represent the values obtained for the PCV2-Systemic Disease herd (PCV2-SD) and grey dots represent the values for the PCV2-Subclinical infection herds (PCV2-SI). Horizontal lines represent log10 mean of PCV2 copies per swab in each category.

In the PCV2-SI herds the percentage of positive samples for each area never reached the values obtained for the PCV2-SD herd. In these herds, the proportion of positive environmental samples detected in each category was: 58.3% in the weaning area, 50.0% in samples from the farm personnel, 43.7% in the farm warehouse, 31.2% in the office, 25.0% in the farm perimeter and 4.2% in the farrowing area. In the gestation area, all the environmental samples from all the PCV2-SI herds were negative to PCV2 DNA, unlike in the PCV2-SD herd. For all the PCV2-SI herds, the mean of PCV2 copies ranged from 2.7 × 10^5^ in the weaning area to 1.6 × 10^3^ in the farm perimeter. Curiously, these results suggest a decreasing trend in the mean of PCV2 copies as we move away from the weaning areas towards other areas without direct contact with the animals (Fig. 1).

All the samples from the gestation area, sow crate, piglet resting area, farrowing air fan and delivery management tool box were only positive in the PCV2-SD herd; in contrast, only two types of samples (pens/computer keyboard and parking area) were negative in the PCV2-SD herd but positive in at least one PCV2-SI herd. The remaining samples tested positive for both PCV2 herd statuses and, except for one environmental sample from Farm C, the amount of PCV2 appeared to be higher in the samples from the PCV2-SD herd (Table 2). It must be noticed that, for both statuses, the warehouse area presented similar levels of PCV2 contamination to other areas which hosted pigs.

## Discussion

Farrow-to-weaning swine farms house animals of different ages, and therefore with different levels of susceptibility to certain agents like PCV2^19^; for this reason, it is essential to work in a well-defined routine so as to avoid the transmission of pathogens among the different production stages. As PCV2 is a ubiquitous virus, it is interesting to know which farm facilities present the highest levels of contamination in order to improve management practices and reduce PCV2 within-farm dissemination. Regarding this, the present work constitutes an approach to the understanding of PCV2 environmental distribution in swine herds and, as far as we know, the first demonstration of PCV2 contamination of farm personnel, management implements and farm facilities without direct contact with pigs.

Our results suggest that the weaning area is the facility with the highest levels of PCV2 environmental contamination for both PCV2 herd statuses. This was expected, since PCV2 infection and therefore viral shedding typically appears at this production stage^14^, which would theoretically favour the accumulation of viral particles in the environment. Regarding PCV2 presentation, it has been described that infection prevalence, viremia and viral shedding are higher in farms with PCV2-SD than in herds without clinical signs^11,13,20^. This is concordant with our results, since the PCV2-SD herd seemed to present more positive environmental samples and higher PCV2 counts than the PCV2-SI herds, not only in the weaning area but also in the rest of dependencies, probably due to the presence of a larger number of infected pigs and higher viremia. However, it must be noted that in one PCV2-SI herd (Farm D) all environmental samples from the weaning area tested negative, despite the existence of viremia in the animals. A possible explanation to this fact may be related to the onset of PCV2 infection in this herd; if the infection were recent, the viral shedding and thus the environmental load would still be low, reducing the probability of PCV2 environmental detection. Nevertheless, regardless of this unexpected event, these results remark the importance of applying effective cleaning and disinfection measures in the weaning area in order to reduce PCV2 environmental load and avoid the possibility of indirect transmission to the following production batch.

With respect to the other farm facilities in direct contact with the animals (gestation and farrowing areas), our results may indicate a potential difference in PCV2 environmental load between both herd statuses, that should be evaluated in further studies. In the PCV2-SD herd, all the environmental samples from the gestation area and almost all from the farrowing area were positive, showing medium-high counts of PCV2 copies. It has been reported that PCV2 can be detected in blood, oral fluids, faeces and other excretions from the sow^18^; therefore, the odds are that the detected PCV2 environmental load came from the animals housed at these facilities, which may also be indicative of an active infection in the sows. In addition, the detection of PCV2 in samples from the farrowing area, like the piglet resting area or delivery management instruments, can have an important implication in the maintenance of the infection in the herd, since the new-born piglets would be exposed to the virus from birth. In contrast with the environmental viral load detected in the gestation and farrowing areas of the PCV2-SD herd, almost all samples taken in these facilities in the PCV2-SI herds were negative (only one positive sample from the farrowing area in one herd). These results suggest that PCV2 shedding in animals from these areas would be very low or even zero, and for this reason the environmental viral load could be practically null in these dependencies. Thus, this hypothesis is coherent with the results of several studies reporting an absence of viremia in farrowing sows and its piglets from PCV-SI herds^21,22^. Besides, it must be noted that the only positive sample in these areas corresponded to a farrowing corridor, whereas other samples in direct contact with the animals tested negative; therefore, a plausible explanation is that viral particles were carried to this area by farm personnel or any contaminated fomite from other farm facilities. Nevertheless, it should be mentioned that it is possible to detect infected animals at this production stage in herds without any PCV2-associated problems^23^, so the possibility of PCV2 contamination coming from the housed animals in these facilities must not be completely ruled out.

Regarding the obtained results from farm workers and their clothing, qPCR analysis showed significant levels of PCV2 contamination, especially in workwear and boots. This is of extreme importance, since the movement of personnel among the different farm facilities during daily routine work may favour PCV2 dissemination from contaminated areas to other farm dependencies. It must be noted that this is not the first demonstration of PCV2 detection in clothing: in a previous study^24^, we reported that it is possible to detect PCV2 in the personal protective equipment (PPE) used by farm visitors just by staying one hour in an PCV2-SI herd, even without coming into direct contact with the animals. However, in the present study samples were taken from staff clothing routinely used in daily farm work. In most farms, workwear and boots are not usually changed every day^25^; for this reason, we sampled these elements in order to have a proper estimation of the viral load that farm workers can carry with them. Therefore, our results warn about the risk of spread of PCV2 within the farm through the movement of the farm’s own personnel, and should be taken into account in disease control programs. For example, some aspects that can be easily implemented or modified to reduce the risk of within-farm PCV2 transmission are the daily change of workwear, the disinfection of work boots (disinfection foot baths, or even the employment of different footwear for each farm facility) and the washing and disinfection of workers´ hands before moving to other farm areas.

qPCR analysis of environmental samples from farm areas without direct contact with the animals (warehouses, offices and perimeter area) also confirmed the presence of considerable levels of contamination with PCV2 DNA. Regarding the warehouses, most samples from floors and different management implements tested positive. Cleaning and disinfection programs in most farms do not usually include the warehouses since, theoretically, these facilities do not have any direct contact with animals. However, as aforementioned, farm personnel may play a key role in the contamination of these premises. Besides, some utensils stored in warehouses, like sorting panels, come into physical contact with pigs or their excretions, being also common their employment through all farm facilities, not only for a particular production stage^26^. Thus, these circumstances could turn warehouses into a critical point for the accumulation of PCV2. In the light of the obtained results, it seems important that the cleaning and disinfection of warehouses is carried out regularly, including also all the materials, utensils and machines stored there. In addition, it may also be advisable to have exclusive utensils for each production stage, as recommended for working footwear. With respect to the other remaining facilities (offices and farm perimeter), our results also showed detectable levels of PCV2 contamination, even with high counts for the PCV2-SD herd. In the office areas, all positive samples corresponded to different elements in contact with people, like tables, chairs or door handles, suggesting that PCV2 was carried there by farm personnel. Thus, cleaning and disinfection protocols should also include the offices and any other facilities which farm workers access regularly. On the other hand, environmental PCV2 detection in the farm perimeter should also be taken into consideration, since it may represent a risk of contamination for visitors or vehicles and, consequently, the possible spread of PCV2 to other farms^27^. Therefore, the adoption of measures like pressure washing of visiting vehicles and posterior disinfection would also be advisable (disinfection gates, wheel baths or similar strategies).

Finally, two aspects concerning the performance of this study must be mentioned. Firstly, the presence of PCV2 DNA in environmental samples does not ensure the infectivity of the viral particles. Nevertheless, the possibility that PCV2 remains viable in the environment must not be ruled out due to its high resistance to adverse conditions^2,3^. However, this study is mainly focused on identifying possible critical points where the virus could accumulate and persist, and therefore the detection of PCV2 DNA should be considered as a risk of infection. Secondly, this study was performed in non-vaccinated commercial swine herds, although nowadays vaccination is a common practice in many farms^8^. However, non-vaccinated herds were chosen in this study to avoid the possible interference of vaccination in PCV2 shedding and consequently in the environmental viral load. This fact limited the number of included herds, making it difficult to study other possible factors that could be influencing the obtained results, like the wide range of positive samples in PCV2-SI herds. Nevertheless, our results represent a useful starting point for further studies, like the assessment of vaccine efficacy in terms of viral shedding reduction and within-farm environmental contamination, as well as the identification of farm factors that may influence the distribution of PCV2 in the different farm areas.

## Conclusion

The present study constitutes the first demonstration of PCV2 contamination of swine farm personnel, management implements and farm facilities without direct contact with the animals. Our results suggest that these elements and areas may play a key role in the maintenance of infection at the farm, as well as having important implications in the within-farm transmission of PCV2. Thus, this work could be helpful for revising the farm management routines to improve the control of PCVDs. In addition, the detection of PCV2 in areas such as farm warehouses, offices and farm perimeter indicates that these facilities must be included in the cleaning and disinfection procedures, since they may represent critical points where PCV2 tends to accumulate, and therefore act as possible sources of infection and/or reinfection. Finally, the levels of viral load detected in animal facilities like the weaning area also highlights the importance of implementing effective cleaning and disinfection measures between consecutive production batches.

## Methods

### Farm selection and classification criteria

To perform this study, farrow-to-weaning swine farms without vaccination against PCV2 were required. In addition, these farms should have a similar census of sows (100–500 animals). Different farm practitioners informed us about possible herds gathering these conditions and, subsequently, we reached out to these farms in order to propose them to be part of the study.

Regarding PCV2 presentation, the farms were classified as PVC2-SD following previously described criteria^28^: (1) an increase of post-weaning mortality and wasting, and (2) postmortem diagnosis of PCV2-SD in at least one out of five pigs (wasting, skin pallor or dyspnoea; moderate to severe lymphocyte depletion of lymphoid tissues; moderate to severe PCV2 amount in those tissues). Farms which did not meet both criteria, but where PCV2 circulation was demonstrated, were classified as PCV2-SI herds. To demonstrate PCV2 circulation, the formula mentioned by Thrusfield was used^29^. Thus, blood samples from 20 piglets at the end of the weaning phase were collected to ensure the detection of a prevalence rate of at least 15%. All methods were carried out in accordance with relevant guidelines and regulations established by the Spanish competent authority (Xunta de Galicia), and the study protocol was approved by the bioethics committee of the University of Santiago de Compostela.

In addition, data of post-weaning mortality was recorded in each farm, necropsies of death pigs were performed and samples from inguinal lymph nodes, spleen and lung were taken for qPCR and histopathological analysis.

### Environmental sampling

Environmental sampling was performed following a swabbing method previously reported to detect virus from livestock surfaces^30^. Table 3 describes the farm areas and the elements sampled in each one (six different farm facilities with and without direct contact with the animals). In addition, samples from the farm staff were included to assess the possible role of workers in the within-farm PCV2 dissemination. Informed consent was obtained from all the individual participants included in the study. Each environmental sample was collected using one dry sterile cotton-tipped swab of 11 mm in diameter, swabbing the surface of the different elements or areas as described in Table 4.

**Table 3:** Categories of environmental samples and samples included in them.

Category	Sample type
Farrowing area (10 days post-farrowing)	Sow feeder, sow crate, piglet resting area, farrowing corridor, farrowing air fan, delivery management tool box.
Weaning area (piglets with 8 weeks old or more)	Piglet hopper, weaning pen wall, weaning pen floor, weaning corridor, pen railing, weaning air fan.
Gestation area	Sow hopper, gestation pen floor, gestation corridor.
Farm warehouse	Warehouse floor, working utensils, feed wagons, pressure washer, sorting panel.
Farm personnel	Farmer hands, nostrils, hair/hat, workwear and work boots.
Office	Office floor, door handles, pens/computer keyboard, tables/chairs.
Perimeter	Parking area, farm main entrance, feed silo rungs.

**Table 4:** Environmental samples and protocol of swabbing to each one.

Environmental sample type	Swabbing protocol (one swab per sample)
Sow feeder, piglet resting area, piglet hopper, pen wall, gestation sow hopper.	In eight different elements of each type, 25 × 25 cm area per element^33^.
Sow crate.	Surface of lateral and rearward lower bars of eight different crates.
Farrowing corridor, weaning corridor, weaning pen floor, gestation pen floor, gestation corridor, warehouse floor, office floor, parking area, farm main entrance.	100 steps with polyethylene boot covers over the surface, and then both boot covers were swabbed as indicated previously^27^: in zigzag from the toe region to the heel.
Farrowing air fan, weaning air fan.	Surface of fan blades or protective grating if blades are not accessible.
Delivery management tool box.	All ventral external surface, 50% of internal surface and all syringes included in it.
Pen railing.	1 m length in zigzag.
Working utensils.	Handle of at least five different utensils (brushes, paddles…) during 10 seconds in each one.
Feed wagons, pressure washer, sorting panel, Tables/Chairs.	50% of the surface.
Farmer hands, nostrils and hair/hat.	As indicated previously^34^: Hands: dorsal and ventral surface of both hands, including each finger and the ventral surface of each fingernail. Nostrils: inserting the swab approximately 1.25 cm in each nostril. Hair/hat: three times around the circumference of the head.
Workwear.	Thorax area, the front and back of each leg from knee to ankle, and the front and back of each arm from elbow to wrist.
Work boots.	As indicated previously^27^: in zigzag from the toe region to the heel.
Door handles.	Surface of exterior and interior door handles of five different doors.
Pens/Computer keyboard.	Surface of computer keyboard or five different pens.
Feed silo rungs.	Vertical and horizontal surface of five different rungs in at least two different silos.

### Laboratory analysis

Blood samples were pooled (five pigs/pool) as it had been previously recommended^31^. DNA isolation was performed from 200 μl of each blood pool using a commercial DNA extraction kit (High Pure PCR Template Preparation Kit, Roche Diagnostics GmbH, Mannheim, Germany) following the manufacturer’s instructions. DNA from tissue samples was extracted following the same protocol using 20 mg of tissue as starting material.

Environmental samples were processed by adding 5 ml of sterile phosphate-buffered saline with 0.05% Tween 20 directly to each tube containing the swab. The tubes were subsequently vortexed for 1 minute and, after 15 minutes of vertical settling, 1 ml of supernatant from each sample was transferred into a sterile Eppendorf tube and kept at −20 °C until DNA isolation was performed. As environmental samples are difficult matrixes due to their high probability of containing PCR inhibitors such as humic acids, the previously recommended analysis scheme^32^ was employed. Briefly, DNA was isolated from 200 μl of swab eluate using firstly a general commercial extraction kit for the complete sample set (High Pure PCR Template Preparation Kit, Roche Diagnostics GmbH, Mannheim, Germany); subsequently, qPCR negative samples for this first DNA isolation were re-extracted again using a commercial extraction kit specifically designed to remove humic acids and other inhibitors (Nucleospin^®^ Soil, Macherey-Nagel GmbH & Co KG, Düren, Germany). In addition, an exogenous internal control (EXOone EXIC, EXOPOL S.L., Zaragoza, Spain) was added to each sample to identify possible qPCR inhibition.

Isolated DNAs from blood, tissues and environmental samples were collected in 100 μl of elution buffer and frozen at −20 °C until qPCR analysis. All DNA samples were analysed by qPCR using a commercial kit targeting the ORF2 gene (EXOone PCV2 oneMIX, EXOPOL S. L., Zaragoza, Spain), following the manufacturer’s instructions. A synthetic DNA positive control supplied with the commercial kit was employed as the positive control and molecular grade water was used as the negative reaction control. Samples were considered positive if Ct ≤ 42 for the PCV2 detection channel. In addition, standard curves were calculated by preparing serial ten-fold dilutions (5 × 10^5^–5 × 10^1^ copies/µl) of the DNA positive control for each plate run. All qPCR reactions were run on an Applied Biosystems ABI Prism 7500 thermocycler (ThermoFisher Scientific, Waltham, USA).
